# Overexpression of EFEMP1 Correlates with Tumor Progression and Poor Prognosis in Human Ovarian Carcinoma

**DOI:** 10.1371/journal.pone.0078783

**Published:** 2013-11-13

**Authors:** Jie Chen, Deying Wei, Yueran Zhao, Xiaoyan Liu, Jie Zhang

**Affiliations:** 1 Department of Maternal and Child Health Care, School of Public Health, Shandong University, Jinan, China; 2 Department of Obstetrics and Gynecology, Shandong Provincial Hospital affiliated to Shandong University, Jinan, China; 3 Department of Obstetrics and Gynecology, Qilu Hospital, Shandong University, Jinan, China; 4 Central Laboratory, Shandong Provincial Hospital affiliated to Shandong University, Jinan, China; Mayo Clinic College of Medicine, United States of America

## Abstract

**Objective:**

This study was to explore the role of EFEMP1 in ovarian tumor progression and its relationship with prognosis of ovarian carcinoma.

**Methods:**

EFEMP1 mRNA and protein expressions in normal ovarian tissue, ovarian tumor, high invasive subclones and low invasive subclones were evaluated by immunohistochemistry and real time RT-PCR. Serum EFEMP1 levels in patients with ovarian tumor were measured by ELISA assay. To assess the angiogenic properties of EFEMP1, VEGF and tumor microvessel density were analyzed in ovarian carcinoma by immunohistochemistry.

**Results:**

EFEMP1 expression was up-regulated in ovarian carcinoma, positively correlated with MVD and VEGF, and its overexpression and high serum levels were significantly associated with high stage, low differentiation, lymph node metastasis and poor prognosis of ovarian cancer. EFEMP1 expression was also found to be over-expressed in the highly invasive subclones compared with the low invasive subclones.

**Conclusion:**

EFEMP1 is a newly identified gene over-expressed in ovarian cancer, associated with poor clinicopathologic features and promotes angiogenesis. This study shows that EFEMP1 may serve as a new prognostic factor and a therapeutic target for patients with ovarian cancer in the future.

## Introduction

Ovarian cancer is one of the most aggressive and heterogeneous cancer types in women and one of the leading causes of gynaecological deaths [1], [2]. Its high mortality is attributable to the fact that the majority of ovarian cancer patients are diagnosed at advanced stages when conventional therapy is less effective [3]. Although substantial advances have been made in ovarian cancer research, the overall 5-year survival rate is still less than 30% [4]. Tumor recurrence and metastasis are considered the major reasons for poor clinical outcome and cancer deaths [5]. Therefore, studying the mechanism of tumor invasion and metastasis will provide further insights into the development and progression of ovarian cancer. In recent years, many biomarkers have been investigated which are involved in the progression of ovarian cancer [6]. But few studies have been done to assess the functions of EFEMP1 in ovarian cancer development.

EFEMP1 (epidermal growth factor–containing fibulin-like extracellular matrix protein 1, fibulin-3) is a member of the fibulin family of extracellular glycoproteins, which are characterized with a fibulin-type C-terminal domain preceded by tandem calciumbinding epidermal growth factor (EGF)-like modules [7]. Fibulins have been shown to modulate cell morphology, growth, adhesion and motility, closely related to a wide variety of cancer development [8]. Overexpressions of fibulin-1 in ovarian cancer and breast cancer have been found to demonstrate oncogenic activity, during these progressions estrogen play important roles [9]–[11]. However in the development of hepatocellular carcinoma, gastric cancer and prostate cancer, fibulin-1 demonstrated tumor-suppressive activity, suppressing tumor growth, enhancing cell apoptosis and inhibiting tumor angiogenesis [12]–[15]. As a tumor suppressor gene, Fibulin-2 inhibited tumor growth, invasion and angiogenesis in hepatocellular carcinoma and breast cancer [16], [17]. In the majority of the development of cancers, Fibulin-5 was widely considered to be associated with the suppression of tumor formation through its control of cell proliferation, motility and angiogenic sprouting [18]–[20].

Paradoxically, EFEMP1 (fibulin-3) can also demonstrate either tumor-suppressive or oncogenic behavior tied to tissue-specific expression. In the research of pancreatic adenocarcinoma, cervical cancer and glioma [21]–[24], increased expression of EFEMP1 has been reported; EFEMP1 plays a role in metastasis and development, and thus links it to poor prognosis. In contrast, a potential cancer-suppressing function of EFEMP1 was found in the study of nasopharyngeal carcinomas, sporadic breast cancer, glioblastoma multiforme, and non-small cell lung cancer (NSCLC), Fibulin-3 was associated with tumour progression and inhibited cell migration and invasion [25]–[28]. But till now, few researches have been made about the relationship between EFEMP1 and ovarian cancer.

The purpose of this study was to assess whether EFEMP1 expression was associated with the prognosis of ovarian cancer, and further to investigate the relation of EFEMP1 expression to angiogenesis.

## Materials and Methods

### Cell lines

The highly invasive subclones (S1, A1) and the low invasive subclones (S21, A19) were isolated from the human ovarian cancer cell lines SKOV3 and 3AO with the limited dilution method. Then, the cell electrophoretic mobility (EPM) of each clone was measured to study charge property using microcapillary electrophoresis (microCE) chips according to Omasu's methods [29]. Finally, MTT assay, Colony formation assay in soft agar, Matrigel invasion assay, Cell migration assay and Tumor xenografts in nude mice were made to confirm that they had high and low metastatic potentials respectively [30], [31]. Cells were cultured in RPMI-1640 supplemented with 10% fetal bovine serum (FBS) and antibiotics (Gibco BRL, Rockville, MD).

### Tissue Specimens

A total of 260 human ovarian tissue specimens were used for this study. Two hundred and twenty (220) epithelial ovarian tumors were obtained from the Department of Gynecology and Obstetrics, Shandong Provincial Hospital between 2005 and 2008. The ovarian tumor specimens included 60 benign ovarian tumors and 160 epithelial ovarian carcinomas (with 58 serous cystadenocarcinoma, 56 mucinous cystadenocarcinoma and 46 endometrioid carcinoma). All of the ovarian cancer patients were clinically staged according to the FIGO staging system [with 74 low stage tumors (FIGO stages I and II) and 86 high stage tumors (FIGO stages III and IV)]. None of the ovarian cancer patients received preoperative radiation or chemotherapy. Forty (40) normal ovary tissue specimens were obtained from the Department of Gynecology and Obstetrics, Shandong Provincial Hospital. The study was approved by the Institutional Medical Ethics Committee of Shandong University.

### Blood samples

Blood samples were accordingly obtained with the written informed consent from the same 160 women with epithelial ovarian cancer (with 58 serous cystadenocarcinoma, 56 mucinous cystadenocarcinoma and 46 endometrioid carcinoma) and from the same 60 women with benign ovarian tumor at the Department of Gynecology and Obstetrics, Shandong Provincial Hospital between 2005 and 2008. None of the ovarian cancer patients received preoperative radiation or chemotherapy. Sixty control blood samples were obtained from age-matched examinees receiving health checks at Shandong Provincial Hospital and showing no history of disease and no abnormalities in laboratory examinations. The study was approved by the Institutional Medical Ethics Committee of Shandong University.

### Enzyme-linked immunosorbent assay

Levels of EFEMP1 in serum samples were measured with sandwich enzyme-linked immunosorbent assay (ELISA) using human EFEMP1 ELISA assay kits (Immuno-Biological Laboratories, Japan). Serum was diluted with EIA buffer (1% BSA, 0.05% Tween 20 in phosphate buffer) and incubated for 2 hour at 37°C. After 4 washes with EIA buffer, horse radish peroxidase-conjugated antibodies were added and incubated for 30 minutes at 4°C. After 4 washes, 100 µL of tetramethyl benzidine solution was added and incubated for 30 minutes at room temperature. The reaction was stopped with 100 µL of 1 N sulfuric acid and measured by ELISA reader at 450 nm.

### Immunohistochemistry (IHC)

According to standard streptavidin-biotin-peroxidase complex procedures, IHC was performed on formalin-fixed, paraffin-embedded sections (5 µm thick) and cell slides fixed in 4% paraformaldehyde. Briefly, after dewaxing, rehydration, and antigen retrieval, the sections were incubated with mouse anti-human EFEMP1 monoclonal antibody (sc-33722, Santa Cruz Biotechnology, Inc.) diluted 1∶50 in PBS. Human cervical cancer paraffin-embedded sections (EFEMP1 positive) were used as positive controls. A negative control was obtained by replacing the primary antibody with normal mouse immunoglobulin (IgG). Positive expression of EFEMP1 protein was defined as the presence of brown granules in the cytoplasm.

### Immunohistochemistry (IHC) analysis

A semiquantitative scoring system based on intensity of staining and distribution of positive cells was used to evaluate EFEMP1 expression. The intensity of EFEMP1 positive staining ranged from 0 to 3 (negative = 0, weak = 1, moderate = 2, or strong = 3) and the percentage of positively stained cells was scored as 0 (0%), 1 (1 to 25%), 2 (26 to 50%), 3 (51 to 75%), and 4 (76 to 100%). The sum of the intensity and percentage score was used as the final staining score (0 to 7). The sum-indexes (−), (+), (++), and (+++) indicated final staining score of 0, 1–3, 4–5, and 6–7, respectively. For statistical analysis, sum-indexes (−) and (+) were defined as low EFEMP1 expression, while sum-indexes (++) and (+++) were defined as high EFEMP1 expression. Each section was independently scored by two pathologists. If an inconsistency occurred, a third pathologist was consulted to achieve consensus.

### Microvessel assessment

The microvascular density (MVD) assessment was evaluated by CD34 immunohistochemical staining of tumor vessels. Any immune-positive single endothelial cell or endothelial cell clusters and microvessels in the tumor was considered to be an individual vessel and counted, as described by Weidner et al. [32]. Peritumoral vascularity, vascularity in areas of necrosis and vessels with thick muscle wall or in a diameter larger than eight erythrocytes, was excluded from the count. The sections were scanned at low power (100×) to select the most vascularized (hot-spots). The microvessels in the hot-spots were counted at power 400× magnifications, and an average count in three hot spots was calculated as MVD. All counts were made by three independent observers who had no knowledge of the corresponding clinicopathologic data.

### Real-time quantitative RT-PCR (q-RT-PCR)

Total RNA was extracted using Trizol reagent (Invitrogen) and reversed transcribed. Quantitative real-time PCR analysis was performed using ABI PRISM 7500 Real-Time PCR System (Applied Biosystems). Each well (20 µl reaction volume) contained 10 µl Power SYBR Green PCR master mix (Applied Biosystems), 1 µl of each primer (5 µmol/l) and 1 ul template. The following primers were used:

EFEMP1 5′- ACCCTTCCCACCGTATCCA -3′

5′- TCTGCTCTACAGTTGTGCGTCC -3′

β-actin 5′-CCACGAAACTACCTTCAACTCCA-3′

5′-GTGATCTCCTTCTGCATCCTGTC-3′

### Statistical analysis

IHC data were analyzed using chi-square test. Measurement data were expressed as the mean ± SE. For comparison of means between two groups, a two-tailed t-test was used and for comparison of means among three groups, one-way ANOVA were used. Survival curve was calculated using the Kaplan-Meier method and the log-rank test. Correlation between the expression of EFEMP1 and VEGF and MVD were analyzed with Pearson correlation coefficient. Statistical analysis was performed using SPSS software version 13.0. P-value<0.05 was considered statistically significant.

## Results

### Expressions of EFEMP1 in human ovarian tissues

**Figure 1:**
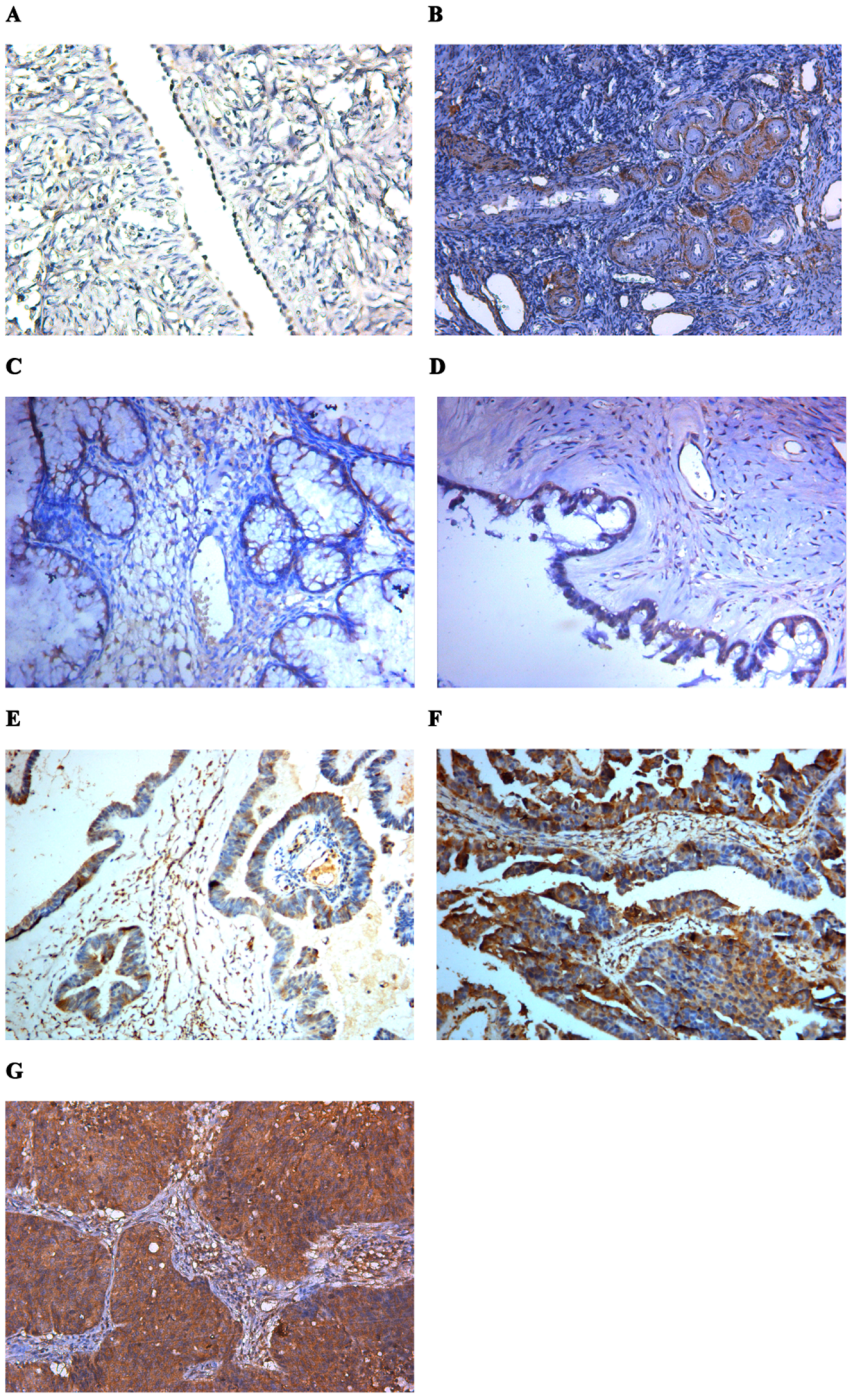
Expression of EFEMP1 in human ovarian tissues. (A) The epithelial cells of normal human ovarian, (B) the stroma of normal human ovarian, (CD) Benign ovarian tumor, (E) High differentiation of ovarian carcinoma, (F) Medium differentiation of ovarian carcinoma, (G) Low differentiation of ovarian carcinoma. (Magnification ×200).

**Figure 2:**
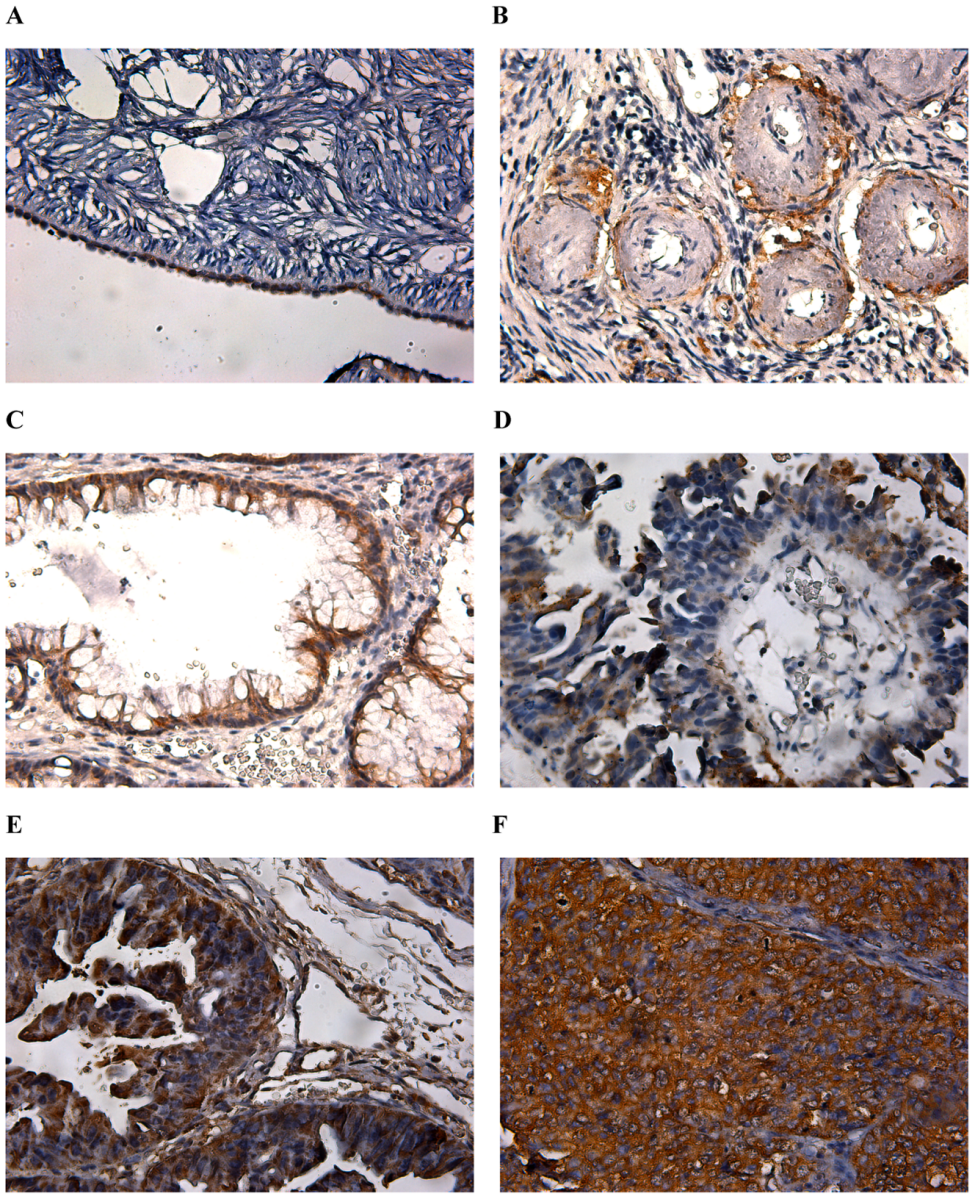
Expression of EFEMP1 in human ovarian tissues with higher-magnification. (A) The epithelial cells of normal human ovarian, (B) the stroma of normal human ovarian, (C) Benign ovarian tumor, (D) High differentiation of ovarian carcinoma, (E) Medium differentiation of ovarian carcinoma, (F) Low differentiation of ovarian carcinoma. (Magnification ×400).

**Figure 3:**
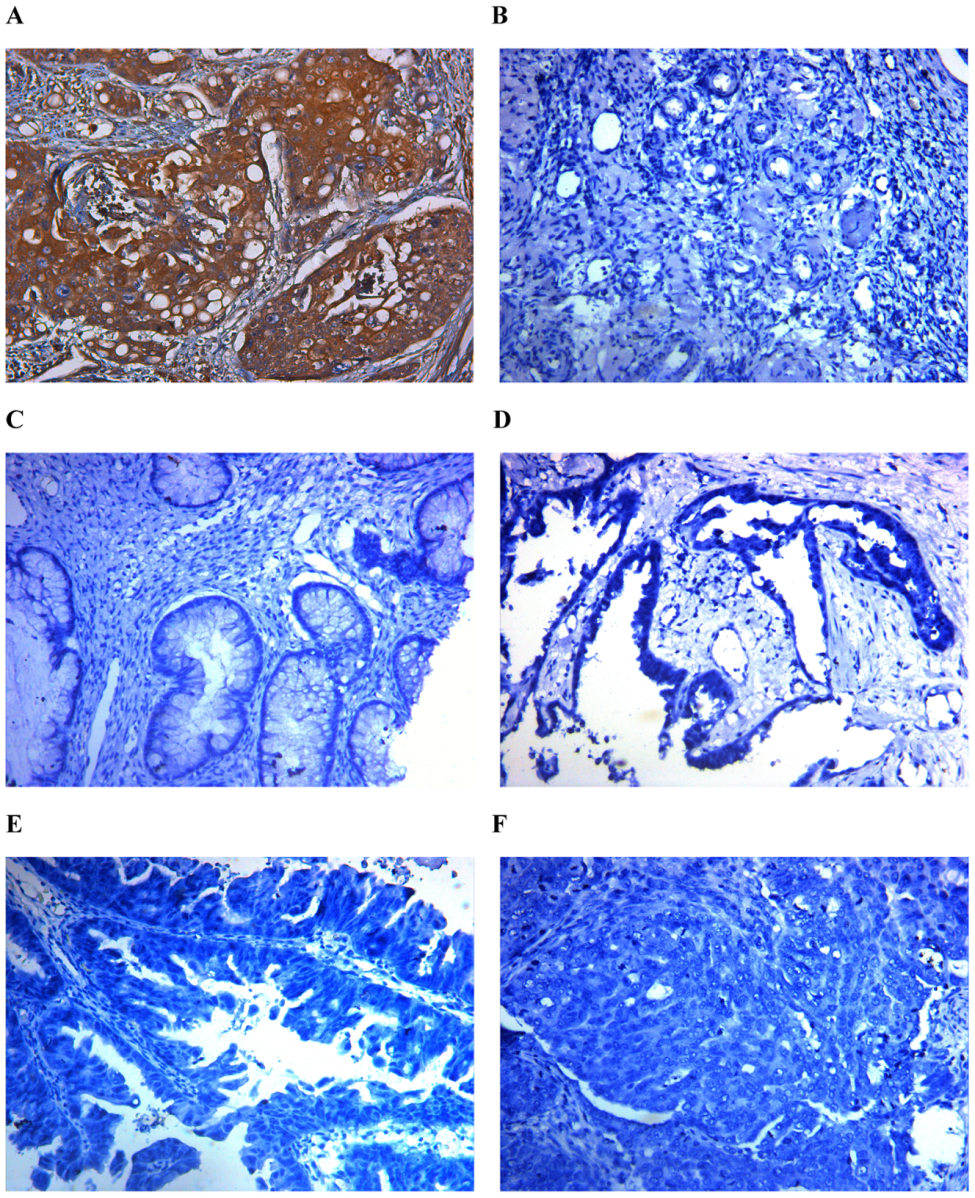
Positive control and negative control of immunohistochemistry (IHC) analysis. Positive control (EFEMP1 positive) (A) Human cervical cancer paraffin-embedded sections. Negative control (B) normal human ovarian tissue, (C) Benign ovarian tumor, (D) High differentiation of ovarian carcinoma, (E) Medium differentiation of ovarian carcinoma, (F) Low differentiation of ovarian carcinoma. (Magnification ×200).

**Figure 4:**
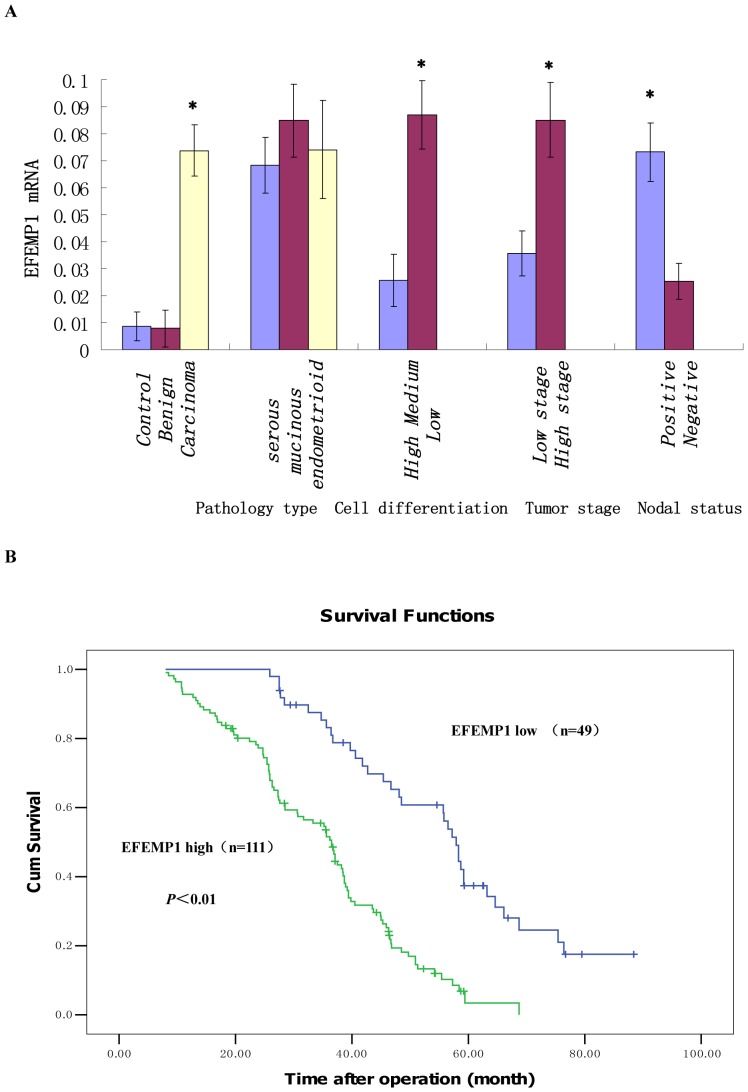
EFEMP1 mRNA expression and Kaplan-Meier analysis. (A) EFEMP1 mRNA expression. High EFEMP1 mRNA expression was also associated with low differentiation, high stage and positive lymph node status of ovarian carcinomas. (B) Kaplan-Meier analysis. Patients with high EFEMP1 expression (blue line) had a much worse prognosis than those with low EFEMP1 expression (green line). *P<0.05 versus control.

To evaluate the mRNA and protein expressions of EFEMP1 in ovarian tissues, we detected 260 human ovarian tissue specimens, including 40 normal human ovaries, 60 benign ovarian tumors and 160 epithelial ovarian carcinomas by immunohistochemistry and real time RT-PCR. As shown in Figure 1 and 2, in normal human ovarian surface epithelial cells, EFEMP1 protein expression was very low (Fig. 1A and Fig. 2A), and in the ovarian stroma, EFEMP1 protein expression was mainly focused around the vasculatures (Fig. 1B and Fig. 2B). However in most ovarian carcinomas, the immunoreactivity was high, and high EFEMP1 protein expression was found in the cytoplasm of ovarian cancer cells (Fig. 1EFG and Fig. 2DEF). To determine the specificity of the antibody, the negative control and positive control was shown in figure 3. Moreover, high EFEMP1 protein expression was associated with low differentiation, high stage and positive lymph node status of ovarian carcinomas (Table 1). Similarly results were also found in real time RT-PCR experiment, EFEMP1 mRNA expression was also very low in normal ovarian tissues and benign ovarian tumors, and significantly enhanced in ovarian carcinoma. Moreover, high EFEMP1 mRNA expression was also associated with low differentiation, high stage and positive lymph node status of ovarian carcinomas (Table 2 and Figure 4A). To evaluate the prognostic value of EFEMP1 in ovarian cancer, we performed survival analysis using Kaplan-Meier analysis. The result showed that patients with high EFEMP1 expression had a much worse prognosis than those with low EFEMP1 expression (log rank, *P*<0.01; Figure 4B).

**Table 1:** Protein expression of EFEMP1 in human ovarian tissues.

	N	EFEMP1 low		EFEMP1 high		*X^2^*	*P*
		(−/+)		(++/+++)			
		n	%	n	%		
Normal	40	38	95%	2	5%	66.01	<0.01
Benign	60	42	70%	18	30%		
Carcinoma	160	49	30.6%	111	69.4%		
Pathology type						0.236	0.889
*serous cystadenocarcinoma*	58	19	32.8%	39	67.2%		
*mucinous cystadenocarcinoma*	56	16	28.6%	40	71.4%		
*endometrioid carcinoma*	46	14	30.4%	32	69.6%		
Cell differentiation						26.921	<0.01
*High and Medium*	88	42	47.7%	46	52.3%		
*Low*	72	7	9.7%	65	90.3%		
Tumor stage						27.837	<0.01
*Low stage*	74	38	51.4%	36	48.6%		
*High stage*	86	11	12.8%	75	87.2%		
Nodal status						35.752	<0.01
*Positive*	83	8	9.6%	75	90.4%		
*Negative*	77	41	53.2%	36	46.8%		

**Table 2:** mRNA expression of EFEMP1 in human ovarian tissues.

	N	EFEMP1 mRNA	*P*
Control	40	0.0087±0.0053	
Benign	60	0.0079±0.0068	>0.05*
Carcinoma	160	0.0738±0.0095	<0.05**
Pathology type			>0.05
*serous cystadenocarcinoma*	58	0.0684±0.0103	
*mucinous cystadenocarcinoma*	56	0.0849±0.0136	
*endometrioid carcinoma*	46	0.0741±0.0181	
Cell differentiation			<0.05
*High and Medium*	88	0.0256±0.0097	
*Low*	72	0.0869±0.0127	
Tumor stage			<0.05
*Low stage*	74	0.0357±0.0083	
*High stage*	86	0.0851±0.0138	
Nodal status			<0.05
*Positive*	83	0.0732±0.0109	
*Negative*	77	0.0254±0.0067	

Note:

* Benign ovarian tumor compared with healthy control, P>0.05;

** Ovarian carcinoma compared with healthy control and benign ovarian tumor, P<0.05.

### Different expression of EFEMP1 in the highly invasive subclone and low invasive subclone

**Figure 5:**
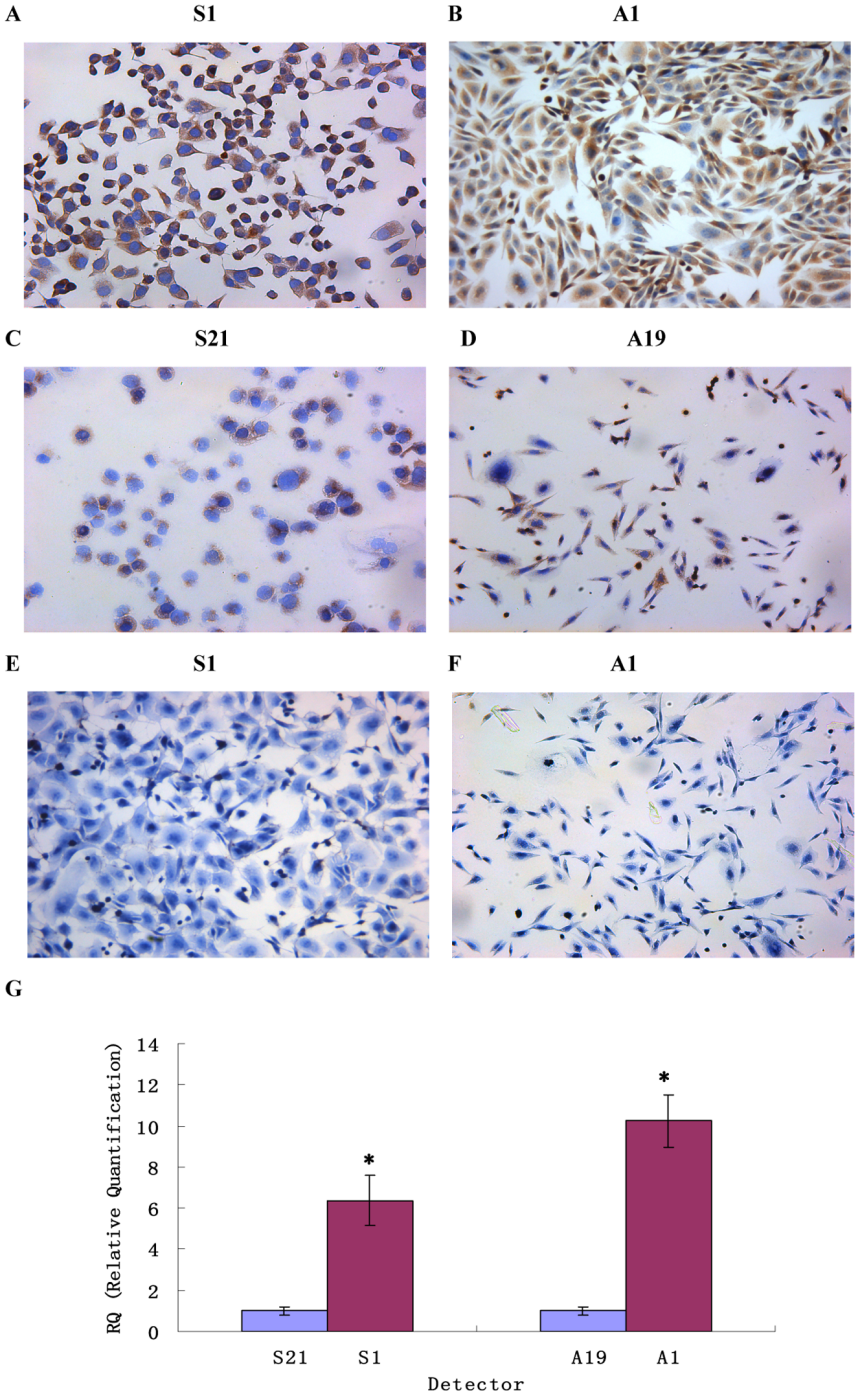
EFEMP1 expression in the highly invasive subclone and the low invasive subclone. (ABCD) EFEMP1 protein expressions of high invasive subclones S1 (A) and A1 (B) and low invasive subclones S21 (C) and A19 (D) as measured by ICC staining. (EF) Negative control of ICC experiment (Magnification ×200). (G) EFEMP1 mRNA expressions of high invasive subclones S1 and A1 and low invasive subclones S21 and A19 as measured by q-RT-PCR. *P<0.05 versus control.

The highly invasive subclone (S1 and A1) and the low invasive subclone (S21 and A19) were derived from the SKOV3 and 3AO human ovarian cancer cell line, by limited dilution method. As shown in Figure 5, EFEMP1 protein and mRNA expressions were very higher in highly invasive subclone (S1 and A1), compared to the low invasive subclone (S21 and A19).

### Serum levels of EFEMP1 in human ovarian tumor and healthy control

**Figure 6:**
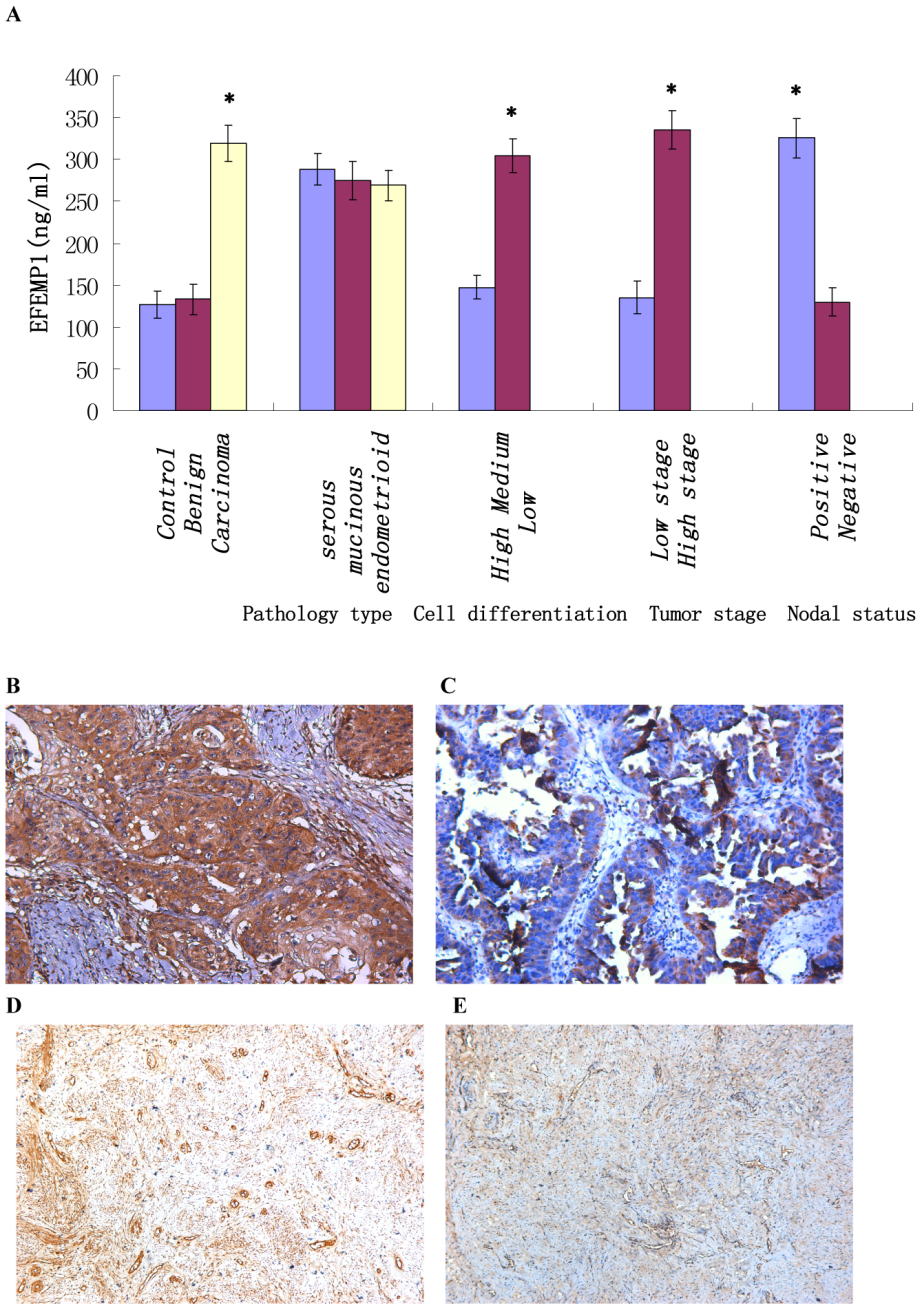
Serum levels of EFEMP1 and immunohistochemical staining of VEGF and CD34 for MVD. (A) Serum levels of EFEMP1. High serum levels of EFEMP1 were associated with low differentiation, high stage and positive lymph node status of ovarian carcinomas. (BC) Immunohistochemical staining of VEGF in low differentiation of ovarian carcinoma (B), and high differentiation of ovarian carcinoma (C) as measured by IHC staining. (Magnification ×200). (DE) Immunohistochemical staining of CD34 for MVD in low differentiation of ovarian carcinoma (D), and high differentiation of ovarian carcinoma (E) as measured by IHC staining. (Magnification ×100). *P<0.05 versus control.

As shown in Table 3 and Figure 6A, the serum EFEMP1 level of ovarian carcinoma was much higher than that of healthy control and benign ovarian tumor (*P*<0.05). No significant difference was found between healthy control and benign ovarian tumor (*P*>0.05). Moreover, high serum levels of EFEMP1 were associated with low differentiation, high stage and positive lymph node status of ovarian carcinomas (*P*<0.05). There were no significant differences among different pathology types of ovarian carcinoma (*P*>0.05).

**Table 3:** Serum levels of EFEMP1 in patients with ovarian tumor.

	N	EFEMP1(ng/ml)	*P*
Control	60	126.47±15.83	
Benign	60	132.76±18.57	>0.05*
Carcinoma	160	319.51±21.65	<0.05**
Pathology type			>0.05
*serous cystadenocarcinoma*	58	287.69±18.81	
*mucinous cystadenocarcinoma*	56	274.53±22.74	
*endometrioid carcinoma*	46	268.79±18.33	
Cell differentiation			<0.05
*High and Medium*	88	147.29±13.88	
*Low*	72	304.37±20.26	
Tumor stage			<0.05
*Low stage*	74	134.73±19.54	
*High stage*	86	335.81±22.69	
Nodal status			<0.05
*Positive*	83	325.49±23.57	
*Negative*	77	129.73±16.44	

Note:

* Benign ovarian tumor compared with healthy control, P>0.05;

** Ovarian carcinoma compared with healthy control and benign ovarian tumor, P<0.05.

### Relationship between EFEMP1 and VEGF and MVD

**Figure 7:**
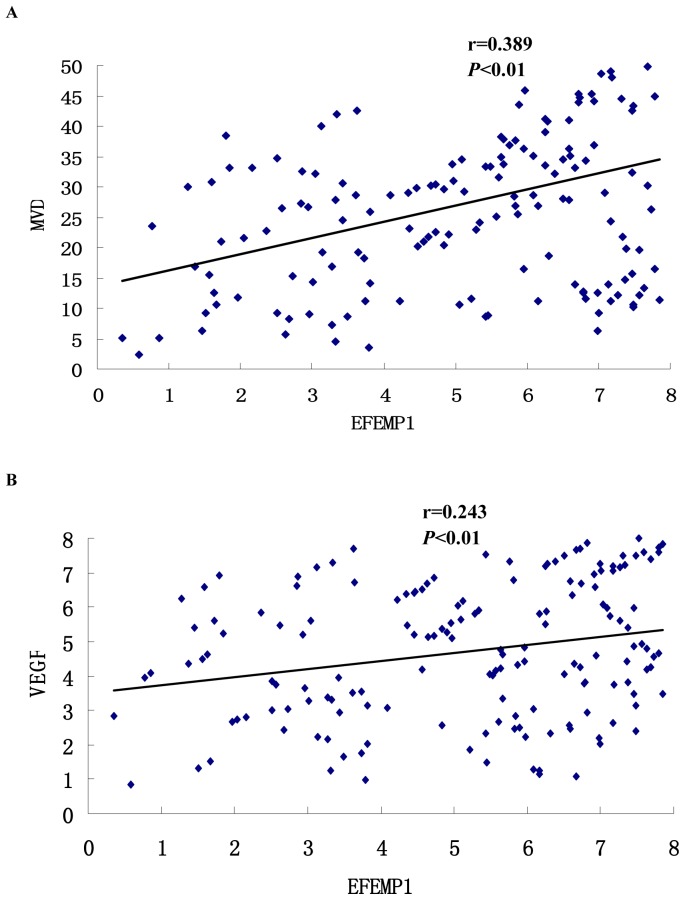
Pearson correlations analysis of EFEMP1 expression with MVD and VEGF. The expression of EFEMP1 positively correlated with MVD (A) and VEGF (B). *P<0.05 versus control.

Figure 6BCDE shows the representative immunohistochemical staining of VEGF and CD34. The immunohistochemical expressions of VEGF and EFEMP1 were evaluated with semiquantitative scoring. Angiogenesis is the process of formation of new microvessels from the preexisting vasculature. Vascular endothelial growth factor (VEGF) is considered as the most potent candidate for the induction of angiogenesis in tumor growth. We want to know whether EFEMP1 is related to angiogenesis, so the Pearson correlation coefficient was calculated to assess the correlation between EFEMP1 and MVD, and the results showed a positive correlation, with high statistical significance (Fig. 7A, r = 0.389, P<0.01). Accordingly, by Pearson correlation coefficient, the correlation between EFEMP1 and VEGF also revealed direct relation with high statistical significance (Fig. 7B, r = 0.243, P<0.01).

## Discussion

In the present study, we have demonstrated for the first time that the expression of EFEMP1 is associated with bad clinicopathologic features, neovascularization and poor prognosis in human ovarian carcinomas.

Our immunohistochemical studies showed that there was an up-regulation of EFEMP1 expression in ovarian carcinoma tissues, compared with normal ovarian tissues and benign ovarian tumors. Real time RT-PCR experiment confirmed the results that the mRNA expression of EFEMP1 was also up-regulated in ovarian carcinoma tissues. Moreover, high EFEMP1 expression was associated with low differentiation, high stage and positive lymph node status of ovarian carcinomas. Similar results also appeared in the research of pancreatic adenocarcinoma, cervical cancer and glioma. In pancreatic adenocarcinoma, EFEMP1 was found significantly up-regulated in the highly metastatic cell line and promoted xenograft formation in vivo [21]. In cervical cancer, EFEMP1 expression was positively correlated with MVD and VEGF, and its over-expression was found to be significantly associated with lymph node metastasis, vascular invasion and poor survival [22]. In gliomas, fibulin-3 was found highly up-regulated in gliomas and cultured glioma cells, overexpression and knockdown experiments revealed that fibulin-3 enhanced substrate-specific cell adhesion and promoted cell motility [24]. In our study, EFEMP1 was over-expressed in ovarian cancer, and associated with poor clinicopathologic features.

We also found that EFEMP1 expression was positively correlated with MVD and the expression of VEGF, which indicated that EFEMP1 may promote angiogenesis. Similar results were found in pancreatic adenocarcinoma and cervical cancer. Seeliger et al. demonstrated that overexpression of EFEMP1 in FG cells, a human pancreatic adenocarcinoma cell line, resulted in a stimulation of VEGF production and an increased number of CD34-positive microvessels in the tumor specimens [21]. Song et al. reported that EFEMP1 gene transfection elevated the VEGF protein level in Hela cells, a cervical cancer cell line, the tumors with EFEMP1 overexpression showed a faster growth rate and had a higher level of VEGF expression and microvascular density [23]. In contrast to our results, EFEMP1 was found to exert antiangiogenesis effect. Albig et al. discovered Fibulin-3 as novel antagonists of endothelial cell activities capable of reducing tumor angiogenesis and, consequently, tumor growth in vivo [33]. Such disparity may be due to the fact that tumor microenvironment influences the tumor genes to promote angiogenesis and metastasis [34]. Of cause, further researches need to be done in the future, including cell transfection experiment, chorioallantoic membrane (CAM) assay and tumor xenografts in nude mice assay to confirm our result.

High serum levels of EFEMP1 were also found in ovarian carcinoma rather than in healthy control and benign ovarian tumor, and associated with low differentiation, high stage and positive lymph node status of ovarian carcinomas. This discovery may aid in determining the diagnosis and prognosis of ovarian carcinoma. Similar result was found in pleural mesothelioma, the plasma fibulin-3 level was significantly elevated in patients with mesothelioma [35]. New biomarker can help to detect ovarian carcinoma at an earlier stage and to individualize treatment strategies.

In conclusion, EFEMP1 is a newly identified gene overexpressed in ovarian cancer, associated with poor prognosis and promotes angiogenesis. Serum levels of EFEMP1 may be helpful to early diagnosis and prognosis judgment. EFEMP1 may serve as a new prognostic factor and a therapeutic target for patients with ovarian cancer in the future.

## References

[1] Jemal A, Siegel R, Xu J, Ward E (2010) Cancer statistics, 2010. CA Cancer J Clin 60: 277–300. 20610543 10.3322/caac.20073

[2] Brun JL, Feyler A, Chene G, Saurel J, Brun G, et al (2000) Long-term results and prognostic factors in patients with epithelial ovarian cancer. Gynecol Oncol 78: 21–7. 10873404 10.1006/gyno.2000.5805

[3] Cho KR, Shih Ie M (2009) Ovarian cancer. Annu Rev Pathol 4: 287–313. 18842102 10.1146/annurev.pathol.4.110807.092246 PMC2679364

[4] Bast RC Jr, Hennessy B, Mills GB (2009) The biology of ovarian cancer: new opportunities for translation. Nat Rev Cancer 9: 415–28. 19461667 10.1038/nrc2644 PMC2814299

[5] Chaffer CL, Weinberg RA (2011) A perspective on cancer cell metastasis. Science 331: 1559–64. 21436443 10.1126/science.1203543

[6] Coticchia CM, Yang J, Moses MA (2008) Ovarian cancer biomarkers: current options and future promise. J Natl Compr Canc Netw 6: 795–802. 18926090 10.6004/jnccn.2008.0059 PMC3381792

[7] de Vega S, Iwamoto T, Yamada Y (2009) Fibulins: multiple roles in matrix structures and tissue functions. Cell Mol Life Sci 66: 1890–1902. 19189051 10.1007/s00018-009-8632-6 PMC11115505

[8] Gallagher WM, Currid CA, Whelan LC (2005) Fibulins and cancer: friend or foe? Trends Mol Med 11: 336–340. 15961345 10.1016/j.molmed.2005.06.001

[9] Moll F, Katsaros D, Lazennec G, Hellio N, Roger P, et al (2002) Estrogen induction and overexpression of fibulin-1C mRNA in ovarian cancer cells. Oncogene 21 (7) 1097–1107. 11850827 10.1038/sj.onc.1205171

[10] Greene LM, Twal WO, Duffy MJ, McDermott EW, Hill AD, et al (2003) Elevated expression and altered processing of fibulin-1 protein in human breast cancer. Br J Cancer 88 (6) 871–878. 12644824 10.1038/sj.bjc.6600802 PMC2377096

[11] Bardin A, Moll F, Margueron R, Delfour C, Chu ML, et al (2005) Transcriptional and posttranscriptional regulation of fibulin-1 by estrogens leads to differential induction of messenger ribonucleic acid variants in ovarian and breast cancer cells. Endocrinology 146 (2) 760–768. 15528301 10.1210/en.2004-1239

[12] Kanda M, Nomoto S, Okamura Y, Hayashi M, Hishida M, et al (2011) Promoter hypermethylation of fibulin 1 gene is associated with tumor progression in hepatocellular carcinoma. Mol Carcinog 50 (8) 571–579. 21268132 10.1002/mc.20735

[13] Cheng YY, Jin H, Liu X, Siu JM, Wong YP, et al (2008) Fibulin 1 is downregulated through promoter hypermethylation in gastric cancer. Br J Cancer 99 (12) 2083–2087. 18985039 10.1038/sj.bjc.6604760 PMC2607230

[14] Wlazlinski A, Engers R, Hoffmann MJ, Hader C, Jung V, et al (2007) Downregulation of several fibulin genes in prostate cancer. Prostate 67 (16) 1770–1780. 17929269 10.1002/pros.20667

[15] Xie L, Palmsten K, MacDonald B, Kieran MW, Potenta S, et al (2008) Basement membrane derived fibulin-1 and fibulin-5 function as angiogenesis inhibitors and suppress tumor growth. Exp Biol Med (Maywood) 233 (2) 155–162. 18222970 10.3181/0706-RM-167

[16] Law EW, Cheung AK, Kashuba VI, Pavlova TV, Zabarovsky ER, et al (2012) Anti-angiogenic and tumor-suppressive roles of candidate tumor-suppressor gene, Fibulin-2, in nasopharyngeal carcinoma. Oncogene 31 (6) 728–738. 21743496 10.1038/onc.2011.272

[17] Yi CH, Smith DJ, West WW, Hollingsworth MA (2007) Loss of fibulin-2 expression is associated with breast cancer progression. Am J Pathol 170 (5) 1535–1545. 17456760 10.2353/ajpath.2007.060478 PMC1854949

[18] Schluterman MK, Chapman SL, Korpanty G, Ozumi K, Fukai T, et al (2010) Loss of fibulin-5 binding to beta1 integrins inhibits tumor growth by increasing the level of ROS. Dis Model Mech 3 (5–6) 333–342. 20197418 10.1242/dmm.003707 PMC2860852

[19] Hu Z, Ai Q, Xu H, Ma X, Li HZ, et al (2011) Fibulin-5 is down-regulated in urothelial carcinoma of bladder and inhibits growth and invasion of human bladder cancer cell line 5637. Urol Oncol 29 (4) 430–435. 19767220 10.1016/j.urolonc.2009.06.004

[20] Yue W, Sun Q, Landreneau R, Wu C, Siegfried JM, et al (2009) Fibulin-5 suppresses lung cancer invasion by inhibiting matrix metalloproteinase-7 expression. Cancer Res 69 (15) 6339–6346. 19584278 10.1158/0008-5472.CAN-09-0398 PMC2719681

[21] Seeliger H, Camaj P, Ischenko I, Kleespies A, De Toni EN, et al (2009) EFEMP1 expression promotes in vivo tumor growth in human pancreatic adenocarcinoma. Mol Cancer Res 7 (2) 189–198. 19208748 10.1158/1541-7786.MCR-08-0132

[22] Song EL, Hou YP, Yu SP, Chen SG, Huang JT, et al (2011) EFEMP1 expression promotes angiogenesis and accelerates the growth of cervical cancer in vivo. Gynecol Oncol 121 (1) 174–180. 21163514 10.1016/j.ygyno.2010.11.004

[23] En-lin S, Sheng-guo C, Hua-qiao W (2010) The expression of EFEMP1 in cervical carcinoma and its relationship with prognosis. Gynecol Oncol 117 (3) 417–422. 20378157 10.1016/j.ygyno.2009.12.016

[24] Hu B, Thirtamara-Rajamani KK, Sim H, Viapiano MS (2009) Fibulin-3 is uniquely upregulated in malignant gliomas and promotes tumor cell motility and invasion. Mol Cancer Res 7 (11) 1756–1770. 19887559 10.1158/1541-7786.MCR-09-0207 PMC3896096

[25] Hwang CF, Chien CY, Huang SC, Yin YF, Huang CC, et al (2010) Fibulin-3 is associated with tumour progression and a poor prognosis in nasopharyngeal carcinomas and inhibits cell migration and invasion via suppressed AKT activity. J Pathol 222 (4) 367–379. 20927779 10.1002/path.2776

[26] Sadr-Nabavi A, Ramser J, Volkmann J, Naehrig J, Wiesmann F, et al (2009) Decreased expression of angiogenesis antagonist EFEMP1 in sporadic breast cancer is caused by aberrant promoter methylation and points to an impact of EFEMP1 as molecular biomarker. Int J Cancer 124 (7) 1727–1735. 19115204 10.1002/ijc.24108

[27] Hu Y, Pioli PD, Siegel E, Zhang Q, Nelson J, et al (2011) EFEMP1 suppresses malignant glioma growth and exerts its action within the tumor extracellular compartment. Mol Cancer 28;10: 123. 10.1186/1476-4598-10-123 PMC3204287 21955618

[28] Kim EJ, Lee SY, Woo MK, Choi SI, Kim TR, et al (2012) Fibulin-3 promoter methylation alters the invasive behavior of non-small cell lung cancer cell lines via MMP-7 and MMP-2 regulation. Int J Oncol 40 (2) 402–8. 21901248 10.3892/ijo.2011.1191

[29] Omasu F, Nakano Y, Ichiki T (2005) Measurement of the electrophoretic mobility of sheep erythrocytes using microcapillary chips. Electrophoresis 26: 1163–1167. 15704247 10.1002/elps.200410182

[30] Chen J, Zhang J, Zhao Y, Li J, Fu M (2009) Integrin beta3 down-regulates invasive features of ovarian cancer cells in SKOV3 cell subclones. J Cancer Res Clin Oncol 135: 909–17. 19104837 10.1007/s00432-008-0526-8 PMC12160189

[31] Chen J, Liu X, Zhang J, Zhao Y (2012) Targeting HMGB1 inhibits ovarian cancer growth and metastasis by lentivirus-mediated RNA interference. J Cell Physiol 227 (11) 3629–38. 22331597 10.1002/jcp.24069

[32] Weidner N, Folkman J, Pozza F, Bevilacqua P, Allred EN, et al (1992) Tumor angiogenesis: a new significant and independent prognostic indicator in early-stage breast carcinoma. J Natl Cancer Inst 84 (24) 1875–87. 1281237 10.1093/jnci/84.24.1875

[33] Albig AR, Neil JR, Schiemann WP (2006) Fibulins 3 and 5 antagonize tumor angiogenesis in vivo. Cancer Res 66 (5) 2621–9. 16510581 10.1158/0008-5472.CAN-04-4096

[34] Chen L, Sun B, Zhang S, Zhao X, He Y, et al (2009) Influence of microenvironments on microcirculation patterns and tumor invasion-related protein expression in melanoma. Oncol Rep 21 (4) 917–23. 19287989 10.3892/or_00000304

[35] Pass HI, Levin SM, Harbut MR, Melamed J, Chiriboga L, et al (2012) Fibulin-3 as a blood and effusion biomarker for pleural mesothelioma. N Engl J Med 367 (15) 1417–27. 23050525 10.1056/NEJMoa1115050 PMC3761217

